# Supplementary material for: A bistable inhibitory optoGPCR for multiplexed optogenetic control of neural circuits
Source: Nat Methods. 2024 May 29;21(7):1275–87. doi: 10.1038/s41592-024-02285-8 (PMC11239505; doi:10.1038/s41592-024-02285-8)
Supplement: Supplementary file 1 — Supplementary results and discussion, Figs. 1–3 and references. [file 41592_2024_2285_MOESM1_ESM.pdf]

nature methods

Supplementary information https://doi.org/10.1038/s41592-024-02285-8

A bistable inhibitory optoGPCR for multiplexed optogenetic control of neural circuits

In the format provided by the authors and unedited

Supplementary Results and Discussion

Encouraged by the inhibitory performance of *Pd*CO in rodent neurons in vitro and in vivo, we tested if *Pd*CO expression can also convey light-mediated inhibition in non-neuronal tissue like cardiomyocytes as well as in neurons of invertebrate model systems.

PdCO in cardiomyocytes

In the heart, multiple GPCR-mediated mechanisms modulate pacemaking activity. Sympathetic stimulation is mediated via β-adrenergic $G\alpha_s$-signaling while parasympathetic stimulation occurs through M2-muscarinic receptor $G\alpha_i$-signaling. Activation of $G\alpha_s$ or $G\alpha_i$ increases or decreases AC activity and cAMP levels, respectively. Elevated cAMP levels activate hyperpolarization-activated cyclic nucleotide-gated (HCN) ion channels leading to faster depolarization and beating frequency. While *Pd*CO does not inhibit AC via $G\alpha_i$ (main text, Fig. 3j-l), it strongly couples to GIRK channels (main text, Fig. 1g-i) and suppresses calcium transients (main text, Fig. 5d-f).

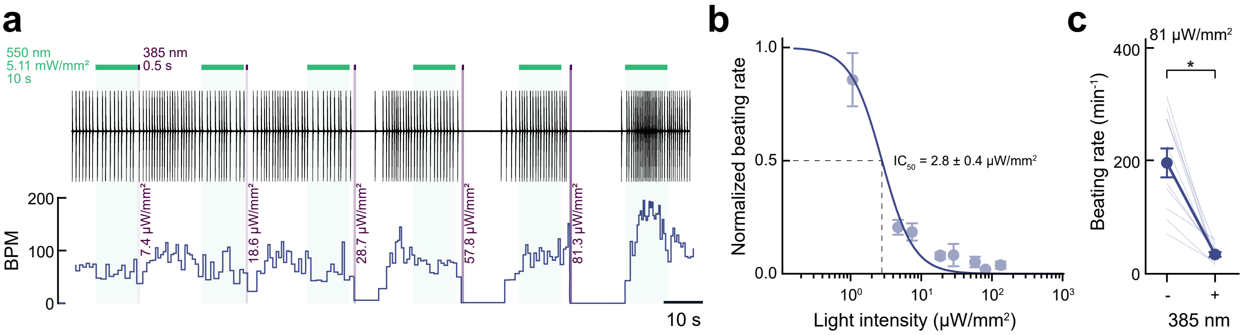

Supplementary Figure 1: *Pd*CO application in cardiomyocytes
a, Top: representative beating trace (one dash per beat) of an atrial cardiomyocyte expressing *Pd*CO, activated with different UV-light intensities and inactivated with green light. Bottom: Analysis of beats per minute (BPM) over time. **b**, Quantification of normalized beating frequency vs applied UV-light intensities from n = 11 different cells. Solid line depicts dose-response fit used for EC_{50} estimation. **c**, Quantification of beating reduction at 81 $\mu W/mm^2$ UV-light compared to non-illuminated conditions (n=11). Statistics: * indicates significance $p<0.05$; t-test (one-sided); p= 5.35E-05. Data is shown as mean ± SEM.

Since atrial cardiomyocytes endogenously express high levels of GIRK channels and rely on calcium influx for their electrical activity, we expressed *Pd*CO in spontaneous active neonatal atrial cardiomyocytes and explored light-mediated suppression of pacing. We found that the spontaneous beating of *Pd*CO-expressing cardiomyocytes could be suppressed after a brief violet light pulse in a light intensity-dependent manner (Supplementary Fig. 1a,b). Inhibition of beating occurred with a half-maximal light dose of 2.8±0.4 µW/mm² (Supplementary Fig. 1b), consistent with similar measurements in neurons. At 81 µW/mm², beating could be efficiently reduced by 80±4% (Supplementary Fig. 1c). These results suggest that *Pd*CO can be efficiently utilized in non-neuronal tissues with comparable efficiency and light sensitivity to neuronal preparations.

Neonatal mouse atrial cardiomyocytes were dissociated from 1-3 day-old CD1 mouse hearts using the Neonatal Heart Dissociation Kit (Miltenyi Biotec) according to the manufacturer's instructions. Cardiomyocytes in suspension (10^6 cells) were transfected by nucleofection (4D-Nucleofector X-Unit, CM-120-150 program, P3 buffer, Lonza) with 5 µg plasmid DNA (pcDNA3.1-CMV-PdCO-mScarlet) and plated on fibronectin coated 24 well plates (µ-Plate ibidi). Video microscopy of beating atrial cardiac cells was performed 3-5 days post transfection in an imaging cell chamber with 5 % CO_2, 80% humidity and 37°C on a Nikon Eclipse Ti2 microscope with an objective S Plan Fl LWD 20x 0.7 NA objective. Changes in spontaneous beating frequency were recorded under IR light using a CMOS camera (Grasshopper3, Teledyne FLIR) and recorded/analyzed online using custom designed software (LabView, National Instruments) as described before[1]. Light stimulation was performed with a LedHUB (Omicron-Laserage) equipped with 385 nm LED (attenuated with a 10 % neutral density filter) and a 555 nm LED. Illumination was controlled by a recording system (PowerLab 4/35 and

LabChart8 Software, AD Instruments). The 385 nm light intensity was varied logarithmically from 0.27 μW/mm² to a maximum of 132.94 μW/mm² every 25 s with a duration of 500 ms. A 10 s 555 nm light pulse was applied before the 385 nm pulse and repeated every 15 s. Light intensities were measured with an S130A power meter sensor at the objective level (Thorlabs).

PdCO in C. elegans

The compact and accessible nervous system of the nematode *C. elegans* enables (optogenetic) perturbations of neural circuits and multicellular systems and therefore can provide valuable insights into the fundamental principles of neurobiology and organismal behavior[2,3]. We therefore explored if *Pd*CO is in principle also applicable to optically manipulate behavior in *C. elegans*.

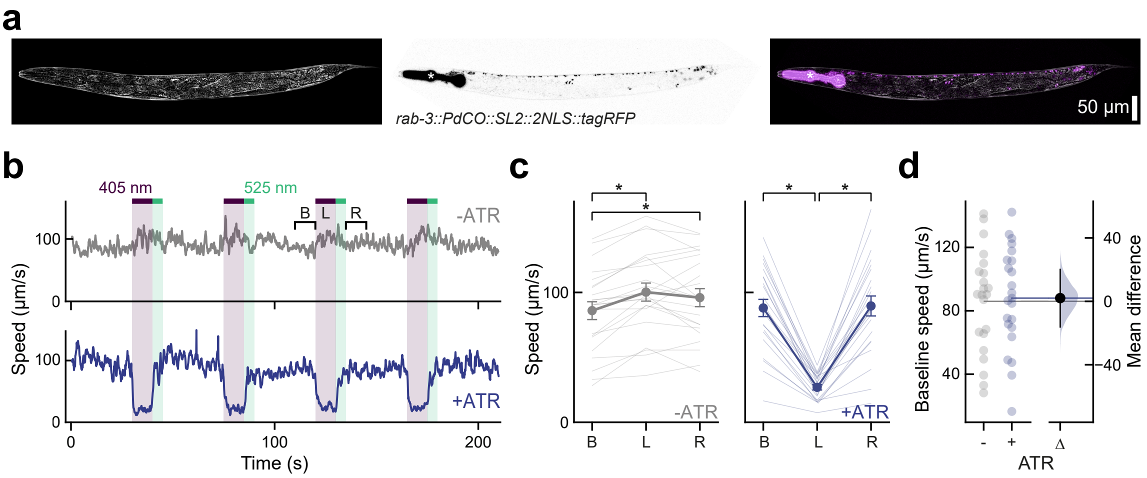

Supplementary Figure 2: *Pd*CO application in *C. elegans*
a, Representative phase contrast (inverted, left) and confocal micrograph of a transgenic worm expressing *Pd*CO and tagRFP pan-neuronally (middle). The overlay is shown to the right. Asterisk marks the expression of the co-injection marker, myo-2::mCherry, in the pharynx. **b**, Mean speed traces of *Pd*CO-expressing worms that were fed with OP50 *E. coli* without all-*trans* retinal (-ATR, top, gray), or with all-*trans* retinal (+ATR, bottom, blue). Purple and green bars and areas depict 405 nm and 525 nm illumination periods, respectively. 10 s intervals for quantification are illustrated for the third illumination cycle: B (baseline), L (light), and R (recovery). **c**, Quantification of average (4 repititions) speed per animal for the three 10 s intervals as shown in b. on the left, data is shown for -ATR animals (gray), while the right plot shows quantification for +ATR animals (blue). Statistics: * indicates significance $p<0.05$; repeated measures one-way ANOVA with Geisser-Greenhouse correction followed by Tukey's multiple comparisons test: p(-ATR; B vs L)<1E-4, p(-ATR; B vs R)=0.0021, p(+ATR; B vs L)<1E-4, p(+ATR; L vs R)<1E-4. Average speed across animals is shown ± SEM and values per animal are shown as thin lines. **d**, The mean difference for the baseline moving speed between -ATR and + ATR animals for each comparison is shown as Cumming estimation plot. Each paired mean difference is plotted as a bootstrap sampling distribution to the left. Mean differences are depicted as dots; 95% confidence intervals are indicated by the ends of the vertical error bars. Number of animals n(-ATR)=22, n(+ATR)=23.

We generated a transgenic *C. elegans* line that expresses codon-optimized *Pd*CO pan-neuronally (Supplementary Fig. 2a) and tested if light application can alter free locomotion. We applied four consecutive illumination protocols, where 405 nm illumination is followed by 525 nm light, thereby activating and deactivating *Pd*CO in an alternating fashion (Supplementary Fig. 2b). While in control nematodes that were not supplied with all-*trans* retinal (-ATR), no reduction of locomotion could be detected, animals fed with retinal (+ATR) showed a 68±2% reduction in moving speed that was fully recovered after green light application (Supplementary Fig. 2b,c). We note that although these animals are *lite-1(ce314)* mutants, and thus are not photosensitive compared to wild-type animals, there is a slight increase in control animals following light stimulation, consistent with previous studies[4]. Baseline moving speeds between +ATR and -ATR fed animals did not differ (Supplementary Fig. 2d), indicating that functional expression in retinal supplied animals does not affect baseline behavior in the dark. Therefore, *Pd*CO might serve as an additional powerful optogenetic tool for studying the circuitry of *C. elegans* and inhibiting synaptic transmission.

To generate a *Pd*CO expression plasmid for *C. elegans* (rab-3p::PdCO::SL2::2XNLS::tagRFP), *Pd*CO was amplified from a plasmid containing a *C. elegans* codon-optimized[5] sequence of *Pd*CO (GenScript), and cloned into pMO40 using Gibson cloning, to generate pAG10.

Worms were maintained according to standard methods. Worms were grown at 200C on nematode growth media (NGM) plates seeded with bacteria (E. coli OP50) as a food source. Strains generated during this study: MOS704 etyEx248 [pAG10 (rab-3p::PdCO::SL2::2XNLS::tagRFP) 50ng/ul; pBS 45ng/ul; myo-2::mCherry 5ng/ul], lite-1(ce314) X. MOS772 syb7630 [etyEx248 integrated], lite-1(ce314) X, outcrossed X2. The transgenic strain carrying the extrachromosomal array was integrated by SunyBiotech.

L4 worms were picked a day before the experiment and transferred to newly seeded plates with 200 µl OP50 (E. coli) that was concentrated 1:10. 100 µM ATR (all-*trans* retinal) was added only to the experimental group's plates. All plates were handled in the dark. The NGM plates that were used for tracking (test plates) were seeded with 40 µl OP50, a day before the experiment, and were left to dry overnight. The worms were transferred to the test plates (~10 worms per plate), ATR and control, and were tracked for 210 s. Animals were tested with 4 consecutive trials of 45 seconds with 30 seconds of baseline, 10 seconds of 405 nm illumination, and 5 seconds of 525 nm illumination. Speed measurements were extracted from WormLab (MBF Bio-science)[6]. The data was analyzed using Python. The speed vector of each individual was smoothed using a median filter (7 frames). For the statistical analysis, the values of all 4 repeats of each time window (10 s) across the recording (prior 405 nm, during 405 nm and post 525 nm) were averaged per individual. A collimated CoolLED PE-4000 served as a light source. Light intensities were 1.43 mW/mm^2 (405 nm) and 0.256 mW/mm^2 (525 nm), measured with a calibrated S170C power sensor (Thorlabs).

For confocal imaging of *C. elegans*, animals were mounted on a 5% agarose pad on a glass slide, on a drop of M9 containing 200 mM sodium azide. A Zeiss LSM 880 confocal microscope was used with 20x magnification.

PdCO in Drosophila larvae

Like *C. elegans*, *Drosophila melanogaster* serves as an important model system to study nervous system function. To test whether *Pd*CO can control circuit function and behavior in *Drosophila melanogaster*, we focused on the larval light avoidance circuit[7–9]. Acute exposure to short wavelength light below 480 nm triggers an aversive stop and turn behavior in larvae. Noxious light can be detected by light sensing cells in the larval head region (Bowlig`s organs) as well as by somatosensory neurons (C4da and V`td2 neurons) lining their body wall (Supplementary Fig. 3a).

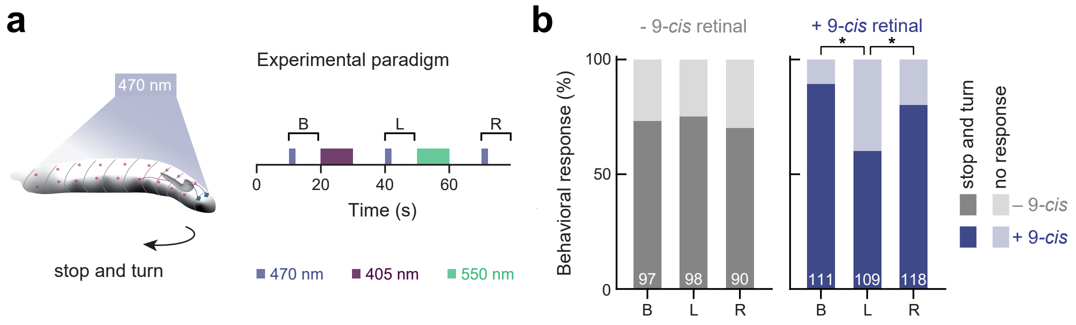

Supplementary Figure 3: *Pd*CO application in cholinergic neurons of *Drosophila* larvae
a, (left) Schematic of *Drosophila* larval light sensing system and avoidance response. Larvae respond to noxious blue light via distinct sensory neurons (indicated in blue and magenta) and display avoidance responses. (right) Experimental paradigm to test the function of *Pd*CO in larval light responses. Behavioral responses to blue light were scored before and after *Pd*CO activation (baseline; B and 405 nm light; L stimulus, respectively), followed by a blue light pulse after inactivation of *Pd*CO with green light (recovery; R stimulus). **d**, (right) *Pd*CO activation in 9-*cis*-retinal fed larvae with 405nm light decreased responses to blue light and could be largely reversed by green light. (left) Control larvae not fed with 9-*cis*-retinal displayed unchanged responses under the same conditions. * indicates significance p<0.05; two-sided X^2- test, p(9-cis B vs L)<0.0001, p(9-cis L vs R)=0.002, number of animals as indicated).

To test if *Pd*CO activation can reversibly suppress this behavior we drove expression in all cholinergic neurons (*cha-Gal4>UAS-Pd*CO) covering the light sensing neurons and additional circuit components. Activation of *Pd*CO in retinal fed larvae led to a 30 % reduction in avoidance responses to blue light, which could be reversed by subsequent inactivation of *Pd*CO by green light, while no difference in behavior was observed in larvae that were not supplied with retinal (Supplementary Fig 3b). *Pd*CO can thus effectively and reversibly inhibit light avoidance behavior in *Drosophila* larvae.

Drosophila melanogaster stocks were maintained at 25^{0}C, 70% humidity on cornmeal/molasses food. *Cha$^{7.4}$-Gal4*[10] was used to drive expression in cholinergic neurons. UAS-PdCO lines were generated by cloning *Pd*CO into pUAST-AttB and using PhiC31-mediated transgenesis[11] in the attP2 site (FlyORF, Zürich, Switzerland).

Larvae expressing *Pd*CO (*UAS-Pd*CO) in cholinergic neurons (*cha$^{7.4}$-Gal4*) were staged in the dark on grape agar plates supplemented with yeast paste with or without 5 mM 9-*cis*-retinal (Sigma-Aldrich). Staged 3rd instar larvae (96 ± 2h AEL) were placed on 1% agar plates under minimum red-light conditions on a frustrated total internal reflection-based tracking system (FIM, University of Münster). Freely moving larvae were stimulated with light of different wavelengths using a CoolLED PE-4000 (CoolLED, Andover, UK). The sequence of light exposure were as followed: no light (10s), blue light (470nm, 1.6-2 mW/mm^{2}, 2-3 s), UV light (405nm, 2.7-3.8 mW/mm^{-2}, 10s), no light, blue light (470nm, 1.6-2 mW/mm^{2}, 2-3 s), green light (550nm, 1.7-2.2 mW/mm^{-2}, 10s), no light(10s), blue light (470nm, 1.6-2 mW/mm^{2}, 2-3 s). A red-light pulse invisible to the animals and not interfering with *Pd*CO activation was used to visualize the blue light stimulus for analysis. Larvae were tracked with 10 frames/s. Between the light stimuli, a period of 10s without light enabled the animals to resume normal locomotion. Behavioral responses were scored when larvae stopped and turned their head in response to blue light.

Supplementary References

1. Bruegmann, T. *et al.* Optogenetic control of heart muscle in vitro and in vivo. *Nature methods* **7**, 897–900 (2010).
2. Husson, S. J., Gottschalk, A. & Leifer, A. M. Optogenetic manipulation of neural activity in C. elegans: From synapse to circuits and behaviour. *Biology of the Cell* **105**, 235–250 (2013).
3. Tsukada, Y. & Mori, I. Optogenetics in Caenorhabditis elegans. in *Optogenetics: Light-Sensing Proteins and Their Applications in Neuroscience and Beyond* (eds. Yawo, H., Kandori, H., Koizumi, A. & Kageyama, R.) 321–334 (Springer, Singapore, 2021). doi:10.1007/978-981-15-8763-4_20.
4. Edwards, S. L. *et al.* A Novel Molecular Solution for Ultraviolet Light Detection in Caenorhabditis elegans. *PLOS Biology* **6**, e198 (2008).
5. Redemann, S. *et al.* Codon adaptation–based control of protein expression in C. elegans. *Nat Methods* **8**, 250–252 (2011).
6. Roussel, N., Sprenger, J., Tappan, S. J. & Glaser, J. R. Robust tracking and quantification of C. elegans body shape and locomotion through coiling, entanglement, and omega bends. *Worm* **3**, e982437 (2014).
7. Keene, A. C. *et al.* Distinct Visual Pathways Mediate Drosophila Larval Light Avoidance and Circadian Clock Entrainment. *J. Neurosci.* **31**, 6527–6534 (2011).
8. Xiang, Y. *et al.* Light-avoidance-mediating photoreceptors tile the Drosophila larval body wall. *Nature* **468**, 921–926 (2010).
9. Imambocus, B. N. *et al.* A neuropeptidergic circuit gates selective escape behavior of Drosophila larvae. *Current Biology* **32**, 149-163.e8 (2022).
10. Kitamoto, T., Xie, X., Wu, C. F. & Salvaterra, P. M. Isolation and characterization of mutants for the vesicular acetylcholine transporter gene in Drosophila melanogaster. *J Neurobiol* **42**, 161–171 (2000).
11. Groth, A. C., Fish, M., Nusse, R. & Calos, M. P. Construction of transgenic Drosophila by using the site-specific integrase from phage phiC31. *Genetics* **166**, 1775–1782 (2004).
