# Supplementary material for: A bistable inhibitory optoGPCR for multiplexed optogenetic control of neural circuits
Source: Nat Methods. 2024 May 29;21(7):1275–87. doi: 10.1038/s41592-024-02285-8 (PMC11239505; doi:10.1038/s41592-024-02285-8)
Supplement: Supplementary file 2 — Reporting Summary [file 41592_2024_2285_MOESM2_ESM.pdf]

nature portfolio

Corresponding author(s): Jonas Wietek, Ofer Yizhar

Last updated by author(s): Mar 1, 2024

Reporting Summary

Nature Portfolio wishes to improve the reproducibility of the work that we publish. This form provides structure for consistency and transparency in reporting. For further information on Nature Portfolio policies, see our Editorial Policies and the Editorial Policy Checklist.

Statistics

For all statistical analyses, confirm that the following items are present in the figure legend, table legend, main text, or Methods section.

n/a	Confirmed	
☐	☒	The exact sample size (*n*) for each experimental group/condition, given as a discrete number and unit of measurement
☐	☒	A statement on whether measurements were taken from distinct samples or whether the same sample was measured repeatedly
☐	☒	The statistical test(s) used AND whether they are one- or two-sided<br>*Only common tests should be described solely by name; describe more complex techniques in the Methods section.*
☒	☐	A description of all covariates tested
☐	☒	A description of any assumptions or corrections, such as tests of normality and adjustment for multiple comparisons
☐	☒	A full description of the statistical parameters including central tendency (e.g. means) or other basic estimates (e.g. regression coefficient) AND variation (e.g. standard deviation) or associated estimates of uncertainty (e.g. confidence intervals)
☐	☒	For null hypothesis testing, the test statistic (e.g. *F*, *t*, *r*) with confidence intervals, effect sizes, degrees of freedom and *P* value noted<br>*Give P values as exact values whenever suitable.*
☒	☐	For Bayesian analysis, information on the choice of priors and Markov chain Monte Carlo settings
☒	☐	For hierarchical and complex designs, identification of the appropriate level for tests and full reporting of outcomes
☒	☐	Estimates of effect sizes (e.g. Cohen's *d*, Pearson's *r*), indicating how they were calculated

Our web collection on statistics for biologists contains articles on many of the points above.

Software and code

Policy information about availability of computer code

Data collection	In vitro electrophysiological data was recorded with Clampex 10.7 (Molecular Devices), WinWCP 5.7 (https://github.com/johndempster/WinWCPXE), Ephus R220 (doi:10.3389/fncir.2010.00100), SutterPatch software (Sutter Instruments), or Patchmaster software (HEKA). Confocal images were acquired using Zeiss Zen 3.7 software (Carl Zeiss), Leica LAS AF software, or Olympus FluoView FV3000. Other imaging data was acquired using Micro-Manager 2.0 (doi:10.14440/jbm.2014.36). Video-microscopy data from cardiomyocytes was aquired with LabView (National Instruments). Bioluminescence data was acquired using MikroWin2010 (Mikrotek Laborsysteme GmbH). Pupil video recordings in anesthetized animals were captured using USB web cameras (Logitech C615) with removed IR filter connected to an TDT RZ2 processor (Tucker Davis Technologies). The RZ2 was programmed through the TDT Synapse software and allowed automated control of laser activation through a MATLAB code, and synchronization with the camera feeds. Nigrostriatal pathway inhibition behavior was video recorded using IC Capture, 30 FPS, 672x672 px. Master 8 controlled the timing of LED protocols and video synchronization signal that was recorded in the video. TTL signals (protocol onset, activation LED on and off, Recovery LED on and off, and video sync on and off, were recorded using OpenEPhys, with their GUI. C. elegans were tracked with WormLab (MBF Bio-science).
Data analysis	Phylogenetic trees were generated with phylogeny.fr (DOI: 10.1093/nar/gkn180). In vitro electrophysiological recordings in cultured cells were analyzed using Clampfit 10.7 (Molecular Devices) as well as IgorPro (Wavemetrics) and NeuroMatic (DOI: 10.3389/fninf.2018.00014) for two-photon experiments. Analysis of mEPSCs data was performed using Easy electrophysiology (v2.3.3b) with a 0.37 correlation cutoff and a 15 pA amplitude threshold due to artificial noise created by series resistance compensation. G-protein coupling assays (TRUPATH, GsX, GloSensor) were analyzed in Microsoft Excel. Confocal imaging and calcium imaging data were analyzed in ImageJ (DOI: 10.1038/nmeth.2019). Data from organotypic slice recordings was analyzed with MATLAB. Atrial cardiomyocyte beating was analyzed using LabView (National Instruments). C. elegans data was analyzed in Python. In vivo experiments were analyzed using Matlab, Graphpad Prism, and DeepLabCut (DOI: 10.1038/s41593-018-0209-y). Statistical analysis was performed with MATLAB, Graphpad Prism 9 or 10 and estimation

statistics were performed online (DOI: 10.1038/s41592-019-0470-3). Schematic brain slice representations were extracted from the enhanced and unified mouse brain atlas (DOI: 10.1038/s41467-019-13057-w).

For manuscripts utilizing custom algorithms or software that are central to the research but not yet described in published literature, software must be made available to editors and reviewers. We strongly encourage code deposition in a community repository (e.g. GitHub). See the Nature Portfolio guidelines for submitting code & software for further information.

Data

Policy information about availability of data

All manuscripts must include a data availability statement. This statement should provide the following information, where applicable:

- Accession codes, unique identifiers, or web links for publicly available datasets
- A description of any restrictions on data availability
- For clinical datasets or third party data, please ensure that the statement adheres to our policy

All data supporting the findings of this study are available as source data files. Raw data will be provided by the corresponding authors upon request.

Human research participants

Policy information about studies involving human research participants and Sex and Gender in Research.

Reporting on sex and gender	N/A
Population characteristics	N/A
Recruitment	N/A
Ethics oversight	N/A

Note that full information on the approval of the study protocol must also be provided in the manuscript.

Field-specific reporting

Please select the one below that is the best fit for your research. If you are not sure, read the appropriate sections before making your selection.

☒ Life sciences ☐ Behavioural & social sciences ☐ Ecological, evolutionary & environmental sciences

For a reference copy of the document with all sections, see nature.com/documents/nr-reporting-summary-flat.pdf

Life sciences study design

All studies must disclose on these points even when the disclosure is negative.

Sample size	Sample sizes were similar to those commonly used in the field (DOI: 10.1126/sciadv.add7729, DOI: 10.1016/j.neuron.2021.03.013, DOI: 10.1016/j.neuron.2021.04.026) and no statistical tests were run to predetermine sample size.
Data exclusions	For autaptic neuron recordings, cells were excluded from analysis if the first EPSC amplitude was below 100pA, and from the analysis of the paired-pulse ratio if optoGPCR activation completely abolished the first EPSC. Further, were cells excluded from analysis if the access resistance was above 20 MΩ or if the holding current exceeded 200 pA. For organotypic slice culture recordings cells were additionally excluded from analysis if a EPSC amplitude drift > 30% occurred.
Replication	All datapoints represent measurements from biological replicates. All in vitro data points originate at least from two independent biological samples (e.g. neuronal cultures or organotypic slices from different batches of mice) or batches of cultured HEK cells. All replication attemps were successful.
Randomization	Randomization was performed in the nigrostriatal pathway silencing experiments, for biophysical characterization of optoGPCRs in autaptic neurons, and for two-photon characterization. Further, the trial order was randomized for stimulation frequency and stimulation location within each experiment for pupillometry experiments. In electrophysiological experiments, cells were always patched randomly without any preselection by fluorescence intensity.
Blinding	Blinding was performed in confocal quantification of expression levels, autaptic benchmark experiments (Fig. 2e) and behavioral experiments silencing the nigrostriatal pathway. Blinding during acquisition was not possible in cases where the cellular expression patterns were clearly different between constructs.

Reporting for specific materials, systems and methods

We require information from authors about some types of materials, experimental systems and methods used in many studies. Here, indicate whether each material, system or method listed is relevant to your study. If you are not sure if a list item applies to your research, read the appropriate section before selecting a response.

Materials & experimental systems

n/a	Involved in the study	
☐	☒	Antibodies
☐	☒	Eukaryotic cell lines
☒	☐	Palaeontology and archaeology
☐	☒	Animals and other organisms
☒	☐	Clinical data
☒	☐	Dual use research of concern

Methods

n/a	Involved in the study	
☒	☐	ChIP-seq
☒	☐	Flow cytometry
☒	☐	MRI-based neuroimaging

Antibodies

Antibodies used

Primary antibodies:
anti-NET 1:300, NET05-2 (MAb Technologies)
guinea pig anti RFP 1:500, 390004 (SYSY)

Secondary antibodies:
AF488 goat anti-mouse 1:500, ab150117 (Abcam)
donkey anti guinea pig CyTM5 1:300, 706-175-148 (Jackson ImmunoResearch)

Validation

Primary antibodies were previously tested in mice (see references at https://www.sysy.com/product-factsheet/SySy_390004 and https://mabtechnologies.com/categories/product/7-norepinephrine-transporter-mouse-net05-2)

Eukaryotic cell lines

Policy information about cell lines and Sex and Gender in Research

Cell line source(s)

HEK293T cells (HEK293T/17, ATCC, #CRL-1573), HEK293 cells stably expressing GIRK1/2 subunits (Dr. A. Tinker, Queen Mary's School of Medicine and Dentistry). HEK293ΔG7 (lacking GNAS/GNAL/GNAQ/GNA11/GNA12/GNA13/GNAZ; A. Inoue, Tohoku University, Japan)

Authentication

Cell lines were authenticated as follows: HEK293T/17 (authenticated by STR profiling by vendor), GIRK1/2 cell line (authenticated by patch-clamp), HEK293ΔG7 (functually tested for the absence of endogenous GS in GsX assay).

Mycoplasma contamination

Cell lines tested negative for mycoplasma contamination.

Commonly misidentified lines
(See ICLAC register)

No commonly misidentified cell lines were used in the study.

Animals and other research organisms

Policy information about studies involving animals; ARRIVE guidelines recommended for reporting animal research, and Sex and Gender in Research

Laboratory animals

In-vivo experiments: DAT-IRES-Cre, age: 8-27 weeks (The Jackson Laboratory, Strain #006660), DBH-Cre, age: 8-12 weeks (B6.FVB(Cg)-Tg(Dbh-cre)KH212Gsat/Mmucd; 036778-UCD-HEMI), pdyn-Cre, age 12-16 weeks (PDYN-IRES-Cre, The Jackson Laboratory, Strain #027958), and WT animals, age 12:-16 weeks (The Jackson Laboratory, Strain #000664).
In-vitro experiments (all age P0): Primary neuronal cultures from Sprague-Dawley rat pups (Envigo), autaptic neuronal cultures from WT mice (C57BL/6NHsd; Envigo, Cat#044), and organotypic slice cultures from Wistar rat pups.
C. elegans (age: larval stage 4): MOS704 etyEx248 [pAG10 (rab-3p::PdCO::SL2::2XNLS::tagRFP), lite-1(ce314) X. MOS772 syb7630 [etyEx248 integrated], lite-1(ce314) X, outcrossed X2.
Drosophila melanogaster (age: staged 3d instar larvae, 96h after egg laying): cha-Gal4>UAS-PdCO

Wild animals

Na wild animals were used in the study.

Reporting on sex

Animals of either sex have been used for all experiments. Sex differences were not considered in study design. Sex based analysis were not performed due to the general molecular nature of GPCR-mediated inhibition of synaptic transmission.

Field-collected samples

No field collected samples were used in the study.

Ethics oversight

All experiments involving animals were carried out according to the guidelines stated in directive 2010/63/EU of the European Parliament on the protection of animals used for scientific purposes. Animal experiments at the Weizmann Institute of Science were approved by the Institutional Animal Care and Use Committee (IACUC) of the Weizmann Institute; experiments in Berlin were approved by the Berlin local authorities and the animal welfare committee of the Charité-Universitätsmedizin Berlin, Germany. Experiments in Bonn and Hamburg were performed in accordance with the guidelines of local authorities. Experiments in Tel Aviv were approved by the Institutional Animal Care and Use Committee (IACUC) of Tel Aviv University (approval 01-19-037). Experiments

performed at the University of Washington, Seattle were approved by the Animal Care and Use Committee of the University of Washington and conformed to US National Institutes of Health guidelines.

Note that full information on the approval of the study protocol must also be provided in the manuscript.
